# Cullin3-TNFAIP1 E3 Ligase Controls Inflammatory Response in Hepatocellular Carcinoma Cells via Ubiquitination of RhoB

**DOI:** 10.3389/fcell.2021.617134

**Published:** 2021-01-21

**Authors:** Yue Liu, Wenjuan Zhang, Shiwen Wang, Lili Cai, Yanyu Jiang, Yongfu Pan, Yupei Liang, Jingrong Xian, Lijun Jia, Lihui Li, Hu Zhao, Yanmei Zhang

**Affiliations:** ^1^Department of Laboratory Medicine, Huadong Hospital Affiliated to Fudan University, Shanghai, China; ^2^Longhua Hospital, Cancer Institute, Shanghai University of Traditional Chinese Medicine, Shanghai, China; ^3^Research Center on Aging and Medicine, Fudan University, Shanghai, China; ^4^Shanghai Key Laboratory of Clinical Geriatric Medicine, Shanghai, China

**Keywords:** RhoB, CRL3s, TNFAIP1, inflammatory response, MAPK signaling

## Abstract

Rho family GTPase RhoB is the critical signaling component controlling the inflammatory response elicited by pro-inflammatory cytokines. However, the underlying mechanisms of RhoB degradation in inflammatory response remain unclear. In this study, for the first time, we identified that TNFAIP1, an adaptor protein of Cullin3 E3 ubiquitin ligases, coordinated with Cullin3 to mediate RhoB degradation through ubiquitin proteasome system. In addition, we demonstrated that downregulation of TNFAIP1 induced the expression of pro-inflammatory cytokines IL-6 and IL-8 in TNFα-stimulated hepatocellular carcinoma cells through the activation of p38/JNK MAPK pathway via blocking RhoB degradation. Our findings revealed a novel mechanism of RhoB degradation and provided a potential strategy for anti-inflammatory intervention of tumors by targeting TNFAIP1-RhoB axis.

## Introduction

Cullin-RING Ligases (CRLs), one major type of E3 ubiquitin ligases, regulate about 20% cellular proteins degradation by ubiquitin-proteasome system (UPS) (Petroski and Deshaies, 2005; Zhao and Sun, 2013). Most of the CRLs consist of Cullin proteins, adaptor proteins, substrate receptor proteins and RING-finger proteins (Cui et al., 2016). Eukaryotic genomes encode eight Cullin proteins (Cullin 1-3, Cullin 4A/4B, Cullin 5, Cullin 7, and Cullin 9) serving as scaffolds of different CRLs (Merlet et al., 2009; Chen and Chen, 2016). The C-terminus of Cullins tightly binds with the Ring finger protein RBX1 or RBX2 which transfers Ub from the Ub-conjugating E2 to the substrates. The N-terminus of Cullins interacts with receptor proteins to recognize specific substrates (Duda et al., 2011). In particular, Cullin3-RING ligases (CRL3s), without receptor proteins, utilize substrates specific Bric-a-Brac/Tramtrack/Broad (BTB) domain proteins to recognize their corresponding substrates and to regulate various biological processes (Kwon et al., 2006; Genschik et al., 2013). Dysregulation of CRL3s leads to tumorigenesis and tumor development (Li et al., 2014; Chen and Chen, 2016; Dubiel et al., 2017). Furthermore, CRL3s play pivotal roles in the innate immune response to infection (Awuh et al., 2015; Dinkova-Kostova et al., 2017). For example, knockdown of the component of Cullin3-Keap1 complex activates NF-κB and drives the expression of pro-inflammatory cytokines (Awuh et al., 2015). However, the regulatory role of CRL3s in inflammatory response has not been fully elucidated.

RhoB, together with RhoA and RhoC, is a member of the Rho family small GTPases that controls numerous essential biological processes, including actin cytoskeleton dynamics, vesicle trafficking, cell cycle and apoptosis (Wheeler and Ridley, 2004; Karlsson et al., 2009; Kim et al., 2009; Li et al., 2011; Vega and Ridley, 2018). As a short-lived protein, the expression of RhoB is induced by a variety of stimuli including epidermal growth factor, UV irradiation, hypoxia and pro-inflammatory cytokines, such as tumor necrosis factor α (TNFα), interferon γ (IFNγ) and interleukin-1 (IL-1) (Fritz and Kaina, 2001; Huang and Prendergast, 2006; Gutierrez et al., 2019). It has been reported that RhoB is the key signaling component in regulating the inflammatory response in endothelial cells and macrophages induced by pro-inflammatory cytokines (Rodriguez et al., 2007; Shi and Wei, 2013; Marcosramiro et al., 2016; Huang et al., 2017). In addition, pro-inflammatory cytokines secreted by tumor cells trigger inflammatory response, which regulate tumor inflammatory microenvironment and promote cancer development and progression (Landskron et al., 2014; Greten and Grivennikov, 2019). However, whether RhoB also regulates the inflammatory response in tumor cells and the underlying mechanisms of RhoB degradation needs to be further explored.

Tumor necrosis factor alpha induced protein 1 (TNFAIP1) is a well-known BTB domain protein that constitutes Cullin3-based ubiquitin ligases, which plays crucial roles in DNA synthesis, apoptosis and cell migration (Chen et al., 2009; Zhu et al., 2014; Liu et al., 2016; Xiao et al., 2020). TNFAIP1 is an immediate-early gene, which is activated by cytokines and chemokines such as TNFα and IL-6 in endothelial cells (Liu et al., 2010; Hu et al., 2012). Recent studies revealed that TNFAIP1 functioned as an inflammatory modulator in Alzheimer’s disease by regulating NF-κB signaling pathway (Zhao et al., 2018). Moreover, TNFAIP1 controls actin cytoskeleton structure and cell movement through mediating the degradation of RhoA (Chen et al., 2009). However, whether RhoB degradation is regulated by TNFAIP1 in inflammatory response remains unknown.

In this study, we demonstrated that Cullin3-TNFAIP1 complex targeted RhoB for ubiquitination and subsequent proteasome-dependent degradation in hepatocellular carcinoma (HCC) cells. Moreover, TNFAIP1 downregulation blocked RhoB degradation, thereby inducing the expression of inflammatory genes IL-6 and IL-8 through activating MAPK signaling pathway upon TNFα stimulation. Our studies revealed a previously unknown mechanism that CRL3 E3 ligases regulating inflammatory response through TNFAIP1-mediated RhoB degradation in HCC cells.

## Materials and Methods

### Cell Culture

HepG2, Huh7, and HEK293T cells were obtained from the Type Culture Collection of the Chinese Academy of Sciences (Shanghai, China), and cultured at 37°C in 5% CO_2_ atmosphere. DMEM medium supplemented with fetal bovine serum (FBS, 10%) and Penicillin-Streptomycin Solution (1%) from Gibco was used for culturing cells.

### RNA Interference

The cells were transfected with siRNA oligonucleotides using Lipofectamine RNAiMAX Transfection Reagent (Invitrogen, United States) according to the manufacture’s instruction. The sequences of siRNA are as follows: for Cullin3, 5′-TTGACGTGAACTGACATCCACATTC-3′ and 5′-TACATATGTGTATACTTTGCGATCC-3′; for TNFAIP1, 5′-TAGAGTAGGACGTTGAGTGTCTCCT-3′ and 5′-CACUC AACGUCCUACUCUATT-3′; for RhoB, 5′-GGCAUUCUCU AAAGCUAUG-3′; for KCTD10, 5′-GAAUGAGCGUCUAA AUCGUTT-3′. All of the above siRNAs were obtained from GenePharma (Shanghai, China).

### Plasmids Construction and Transfection

To generate Flag-RhoB, HA-TNFAIP1, Myc-Cullin3 and His-Ub constructs, Human RhoB, TNFAIP1, Cullin3, and Ub were amplified by PCR and cloned into the modified pCMV-Tag2B, pCMV-HA, pCMV-Myc and pCMV-His vector, respectively. All constructs were verified by sequence analysis. Plasmids transfection were carried out using Lipofectamine 2000 (Invitrogen, United States) following the manufacturer’s instructions.

### Reagents and Antibodies

Recombinant human TNFα was from Beyotime Biotechnology. MG132 and cycloheximide (CHX) were from sigma. The cell lysates for immunoblotting analysis used antibodies against rabbit Cullin3 (Cell Signaling Technology, United States), mouse β-actin (Cwbiotech, China), mouse RhoB (Santa Cruz, United States), rabbit Ubiquitin (Cell Signaling Technology, United States), mouse TNFAIP1 (Santa Cruz, United States), rabbit KCTD10 (Proteintech, United States), mouse Flag (Abmart, China), rabbit Flag (Huabio, China), rabbit Myc (Huabio, China), rabbit HA (Huabio, China), rabbit p-p38 (Cell Signaling Technology, United States), rabbit p38 (Cell Signaling Technology, United States), rabbit p-JNK (Cell Signaling Technology, United States), and rabbit JNK (Cell Signaling Technology, United States). Densitometric analysis of the band intensities was performed using ImageJ.

### Endogenous Immunoprecipitation Assay

Immunoprecipitation of endogenous RhoB or TNFAIP1 was performed with mouse RhoB antibody and mouse TNFAIP1 antibody (Santa Cruz, United States), respectively. Before lysis, the cells were treated with MG132 (Sigma-Aldrich, United States) for 6 h. Five hundred microliter lysis buffer (Thermo Fisher Scientific, United States) was added into dishes. The cells were collected into tube and spun at 13,000 rpm for 15 min. The supernatant was transferred to new tube and incubated with 1 μg RhoB antibody or TNFAIP1 antibody overnight at 4°C. Complexes were pulled down by incubation with protein A/G (Santa Cruz, United States) for another 2 h. The immunoprecipitate was washed 3 times with lysis buffer and analyzed with SDS-PAGE.

### Exogenous Immunoprecipitation Assay

HEK293T cells were transfected with Flag-RhoB and HA-TNFAIP1. After 24 h, the cells were pretreated with MG132 (Sigma-Aldrich, United States) for 6 h. A 500 μL lysis buffer (Thermo Fisher Scientific, United States) was added into dishes. The cells were collected into tube and spun at 13,000 rpm for 15 min. The supernatant was transferred to new tube and incubated with anti-Flag M2 beads (Santa Cruz, United States) overnight at 4°C. The immunoprecipitate was washed 3 times with lysis buffer and analyzed with SDS-PAGE.

### Immunofluorescence

To detect endogenous RhoB and TNFAIP1 colocalization, HepG2 and HEK293T cells were seeded into 3 mm plates with glass slide. After 24 h, the cells were fixed with 4% paraformaldehyde for 20 min. Upon fixation, cells were treated with 0.1% Triton X-100 for 20 min and unspecific staining was blocked by 2% BSA in PBS for 1 h. Cells were incubated with primary antibodies overnight at 4°C. Cells were washed 3 times with PBS and incubated with secondary antibody for 1 h. Secondary antibodies used in this study were goat anti-mouse Alexa Fluor 488 and goat anti-rabbit Alexa Fluor 647 (Invitrogen, United States). Hoechst (Invitrogen, United States) was used to stain nuclei. The plates were analyzed using confocal microscope Leica SP9.

### Ubiquitination Assay

For RhoB ubiquitination analysis, HEK293T cells were transfected with plasmids expressing RhoB, Ub, and TNFAIP1 or Cullin3. The cells were pretreated with MG132 (Sigma-Aldrich, United States) for 6 h. Lyse cells with 100 μL cell lysis buffer (Thermo Fisher Scientific, United States) with 1% SDS (Sangon biotech, China) per plate. Cell lysates were boiled for 10 min. Then diluted in 10 volumes of lysis buffer without SDS. Samples were subjected to immunoprecipitation with anti-Flag M2 beads (Sigma-Aldrich, United States) and immunoblotting with anti-ubiquitin antibody. To detect endogenous RhoB ubiquitination, the cells were transfected with siRNA oligonucleotide. After 96 h post-transfection, the cells were treated with MG132 for 6 h and subjected to immunoprecipitation with RhoB antibody and immunoblotting with ubiquitin.

### RNA Extraction and qPCR

Total RNA was isolated using the Trizol reagent (Invitrogen, United States). The reverse transcription reaction was performed on 1 μg of total RNA per sample using the PrimerScript reverse transcription reagent kit (TaKaRa, Japan) according to the manufacturer’s protocol. After the RT reaction, the quantitative polymerase chain reaction was performed using the Power SYBR Green PCR MasterMix (Applied Biosystems, CA) and specific PCR primers on the ABI 7500 thermocycler (Applied Biosystems) according to the instrument manual. For each sample, the mRNA abundance was normalized to the amount of β-actin. Primers were as follows:

for TNFAIP1, F: 5′-ACCTCCGAGATGACACCATCA-3′,

R: 5′-GGCACTCTGGCACATATTCAC-3′,

for RhoB, F: 5′-CTGCTGATCGTGTTCAGTAAGG-3′,

R: 5′-TCAATGTCGGCCACATAGTTC-3′

for IL-6, F:5′- ACTCACCTCTTCAGAACGAATTG-3′,

R: 5′- CCATCTTTGGAAGGTTCAGGTTG-3′,

for IL-8, F:5′- TTTTGCCAAGGAGTGCTAAAGA-3′,

R: 5′- AACCCTCTGCACCCAGTTTTC-3′,

for β-actin, F:5′- CATGTACGTTGCTATCCAGGC-3′,

R: 5′- CTCCTTAATGTCACGCACGAT-3′.

### Statistical Analysis

The statistical significance of differences between groups was assessed using the GraphPad Prism5 software (GraphPad Software, Inc., San Diego, CA, United States). For comparison of two groups of samples, the two-tailed Student’s *t*-test was used. *P* < 0.05 was considered a statistically significant change (^∗^*P* < 0.05, ^∗∗^*P* < 0.01, ^∗∗∗^*P* < 0.001, ^##^*P* < 0.01, ^###^*P* < 0.001, n.s. = not significant).

## Results

### CRL3s Regulates the Degradation of RhoB

**FIGURE 1:**
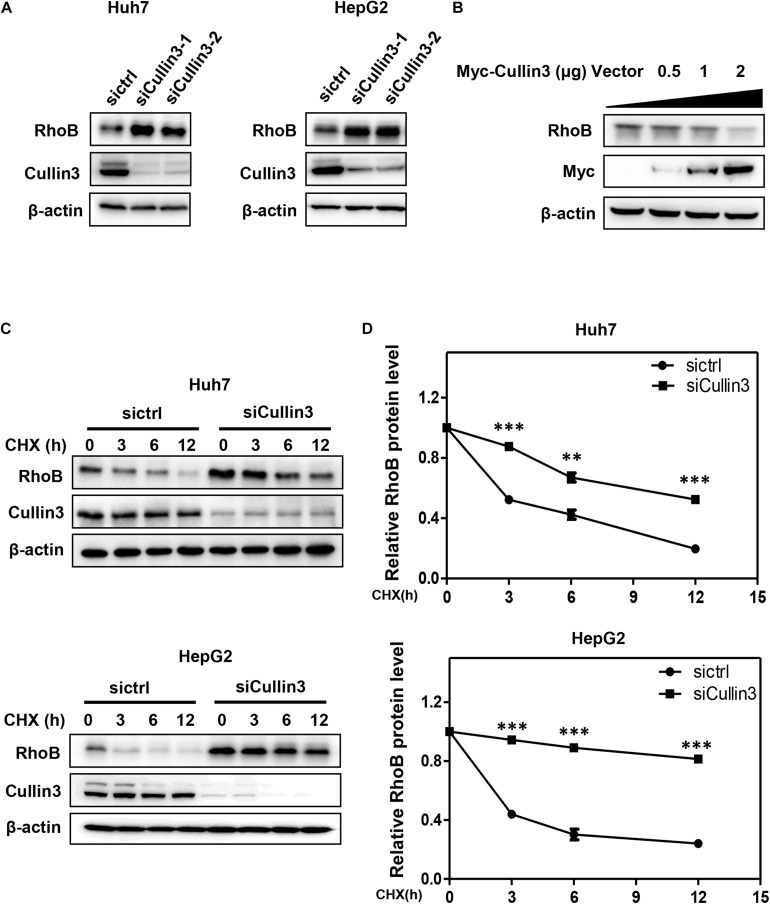
Cullin3 regulated protein degradation of RhoB. **(A)** Downregulation of Cullin3 induced remarkable RhoB accumulation in both Huh7 and HepG2 cells. Cells were transfected with ctrl (control), Cullin3-1 or Cullin3-2 siRNA for 96 h, and cell lysates were harvested for western blot analysis. **(B)** Cullin3 overexpression decreased the protein levels of endogenous RhoB. HEK293T cells were transfected with indicated concentration of Myc-Cullin3 plasmid or empty vector, and cell lysates were harvested for western blot analysis. **(C)** Knockdown of Cullin3 extended the half-life of RhoB. Cells were transfected with indicated siRNAs then treated with 50 μg/mL cycloheximide (CHX) for the indicated time and harvested for western blot analysis. **(D)** The protein levels were quantified by densitometric analysis and statistical analysis was performed. ^∗∗^*P* < 0.01 and ^∗∗∗^*P* < 0.001 vs. control group.

To determine whether RhoB served as a substrate of CRL3s, we first detected the protein level of RhoB upon downregulation of Cullin3 in HCC cells. We found that knockdown of Cullin3 using siRNA induced remarkable RhoB accumulation in both Huh7 and HepG2 cells (Figure 1A). Furthermore, we showed that the RhoB expression were suppressed by overexpression of Myc-Cullin3 in a dose-dependent manner (Figure 1B). Next, the RhoB protein stability in Cullin3-silenced cells was measured in the presence of cycloheximide (CHX), which was applied to impede protein synthesis. The results showed that downregulation of Cullin3 dramatically extended the half-life of RhoB (Figures 1C,D). Taken together, our data indicated that Cullin3 targets RhoB for degradation.

### TNFAIP1 Functioned as the Adaptor Protein for CRL3s to Mediate RhoB Degradation

**FIGURE 2:**
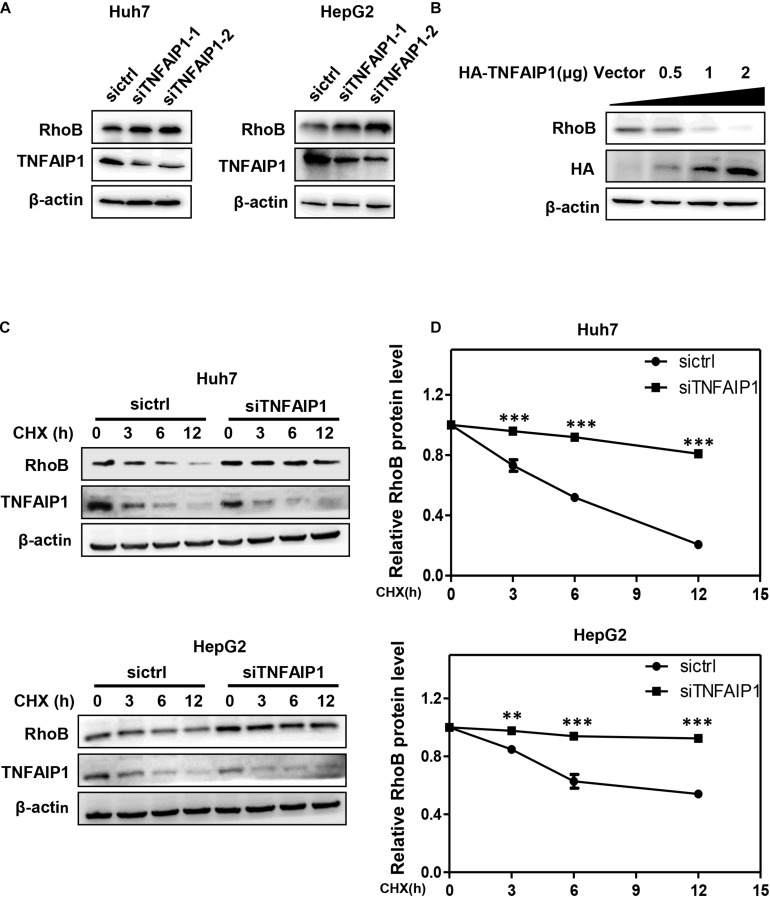
The BTB domain protein TNFAIP1 regulated RhoB degradation. **(A)** Knockdown of TNFAIP1 led to RhoB accumulation in both Huh7 and HepG2 cells. Cells were transfected with ctrl (control), TNFAIP1-1 or TNFAIP1-2 siRNA for 96 h, and cell lysates were harvested for western blot analysis. **(B)** Overexpression of TNFAIP1 down-regulated the protein levels of endogenous RhoB. HEK293T cells were transfected with indicated concentration of HA-TNFAIP1 plasmid or empty vector, and cell lysates were harvested for western blot analysis. **(C)** Ablation of TNFAIP1 prolonged the half-life of RhoB. Huh7 and HepG2 cells were transfected with indicated siRNAs for 96 h. After 50 μg/mL cycloheximide (CHX) treatment at the indicated time, cell lysates were harvested for western blot analysis. **(D)** The protein levels were quantified by densitometric analysis and statistical analysis was performed. ^∗∗^*P* < 0.01 and ^∗∗∗^*P* < 0.001 vs. control group.

Previous studies demonstrated that TNFAIP1 mediated RhoA degradation and controlled actin cytoskeleton structure in Hela cells (Chen et al., 2009). We determined whether TNFAIP1 also regulated the protein level of RhoB. Two pairs of siRNAs specifically against TNFAIP1 were transfected into Huh7 and HepG2 cells. As shown, ablation of TNFAIP1 induced the accumulation of RhoB in Huh7 and HepG2 cells (Figure 2A), while did not affect the expression of Cullin3 (Supplementary Figure 1). In contrast, overexpression of HA-TNFAIP1 promoted the RhoB degradation in a dose-dependent manner (Figure 2B). To further investigate whether TNFAIP1 promote RhoB degradation, we applied CHX to block protein translation and determined the turnover rate of RhoB. We found that knockdown of TNFAIP1 using siRNA significantly extended the half-life of RhoB in both Huh7 and HepG2 cells (Figures 2C,D). These data suggested that TNFAIP1 regulated RhoB degradation.

### TNFAIP1 Interacted With RhoB

**FIGURE 3:**
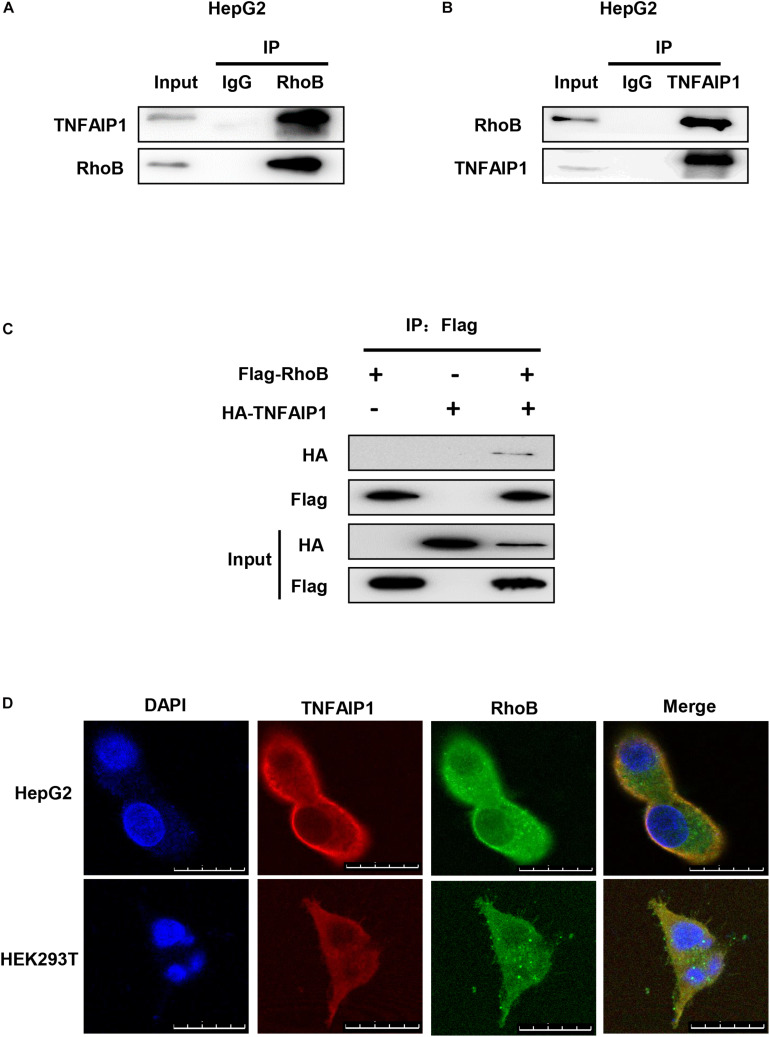
TNFAIP1 interacted with RhoB. **(A)** Endogenous RhoB interacted with TNFAIP1. HepG2 cells were treated with MG132 (5 μM) for 6 h, then cells lysates were subjected to immunoprecipitation with anti-RhoB antibody. **(B)** Endogenous TNFAIP1 interacted with RhoB. HepG2 cells were treated with MG132 (5 μM) for 6 h, then cell lysates were subjected to immunoprecipitation with anti-TNFAIP1 antibody. **(C)** Exogenous RhoB and TNFAIP1 bound to each other. Plasmids expressed Flag-RhoB or HA-TNFAIP1 alone or expressed Flag-RhoB and HA-TNFAIP1 simultaneously were transfected into HEK293T cells. Cell lysates were subjected to immunoprecipitation with anti-Flag M2 beads. **(D)** Endogenous TNAFIP1 and RhoB co-localized in cytoplasm and plasma membrane. HepG2 and HEK293T cells were fixed and stained for TNFAIP1 and RhoB. Bars, 25 μm.

To determine whether TNFAIP1, a well-defined adaptor protein of CRL3 complex, interacted with RhoB, co-immunoprecipitation assay was applied. First, immunoprecipitation assay with anti-RhoB antibody was performed. As shown in Figure 3A, endogenous RhoB could specifically pull-down TNFAIP1 in HepG2 cells. Furthermore, endogenous TNFAIP1 specifically combined with RhoB (Figure 3B). Consistently, overexpression of HA-TNFAIP1 in transfected HEK293T cells also interacted with Flag-RhoB (Figure 3C). Next, we used immunofluorescence assay to determine whether RhoB co-localize with TNFAIP1 in cells, and found that the endogenous protein RhoB co-localized with TNFAIP1 in the cytoplasm and plasma membrane of both HepG2 and HEK293T cells (Figure 3D). Taken together, our data demonstrated that TNFAIP1 specifically bound with RhoB.

### The Ubiquitination of RhoB Was Mediated by Cullin3-TNFAIP1 Complex

**FIGURE 4:**
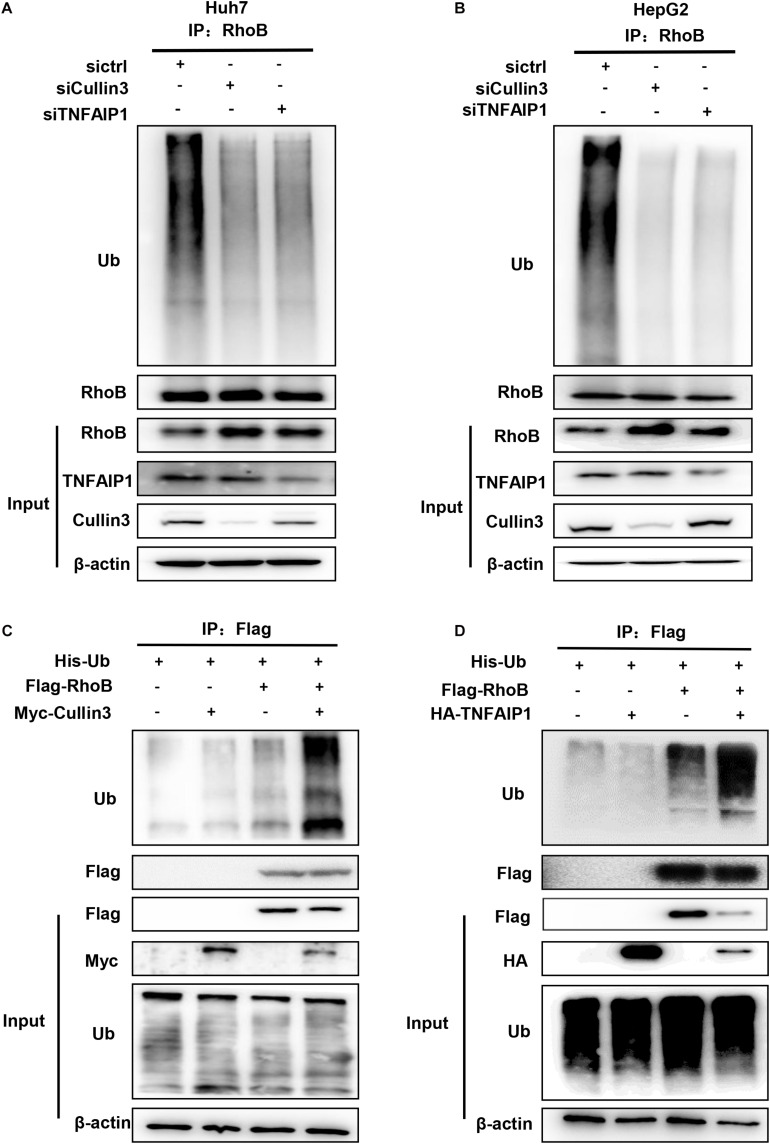
Component of CRL3 mediated RhoB ubiquitination. **(A)** Downregulation of Cullin3 and TNFAIP1 inhibited RhoB polyubiquitination in Huh7 cells. Huh7 cells were transfected with ctrl (control), Cullin3 or TNFAIP1 siRNA for 96 h and followed with MG132 (5 μM) for 6 h. Cell lysates were subjected to immunoprecipitation with anti-RhoB antibody. **(B)** Cullin3 and TNFAIP1 depletion suppressed ubiquitination of RhoB in HepG2 cells. HepG2 cells were transfected with ctrl (control), Cullin3 or TNFAIP1 siRNA for 96 h and followed with MG132 (5 μM) treatment. Cell lysates were harvested and subjected to immunoprecipitation with anti-RhoB antibody. **(C)** Cullin3 promoted RhoB ubiquitination. HEK293T cells were transfected with indicated plasmids combinations of His-tagged Ub (His-Ub), Flag-tagged RhoB (Flag-RhoB), and Myc-tagged Cullin3 (Myc-Cullin3). Cell lysates were subjected to immunoprecipitation assay with anti-Flag beads. **(D)** TNFAIP1 promoted RhoB ubiquitination. HEK293T cells were transfected with indicated plasmids combinations of His-tagged Ub (His-Ub), Flag-tagged RhoB (Flag-RhoB), and HA-tagged TNFAIP1 (HA-TNFAIP1). Cell lysates were subjected to immunoprecipitation assay with anti-Flag beads.

Next, we determine whether downregulation of Cullin3 and TNFAIP1 affected RhoB ubiquitination. As shown in Figures 4A,B, downregulation of Cullin3 or TNFAIP1 via siRNA silencing significantly reduced the ubiquitination level of RhoB in both Huh7 and HepG2 cells. Furthermore, the ubiquitination level of RhoB was also strongly promoted upon Cullin3 or TNFAIP1 overexpression in HEK293T cells (Figures 4C,D). These findings demonstrated that Cullin3-TNFAIP1 complex functioned as an E3 ubiquitin ligase to mediate RhoB degradation.

### Downregulation of TNFAIP1 Enhanced Inflammatory Response via Blocking the Degradation of RhoB Upon TNFα Stimulation

**FIGURE 5:**
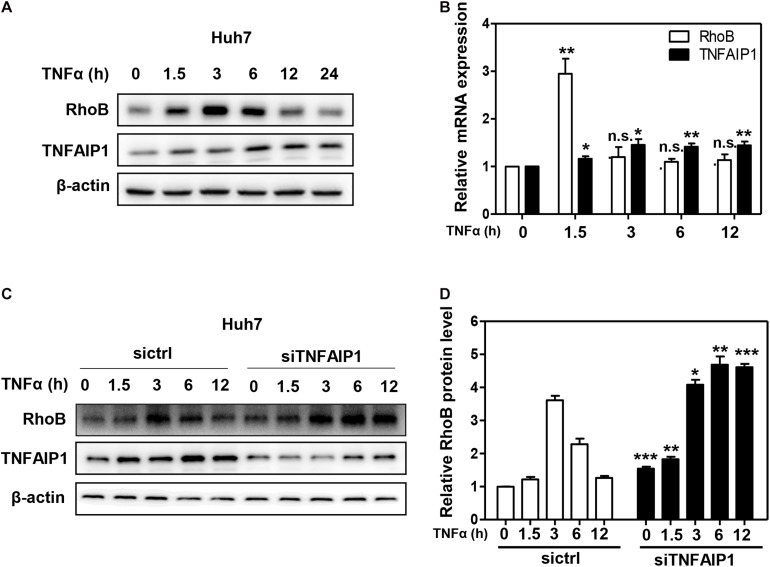
The turnover of RhoB upon TNFα stimulation is regulated by TNFAIP1. **(A)** Time course of induction of TNFAIP1 and RhoB protein levels upon TNFα treatment in Huh7 cells. Huh7 cells were treated with 20 ng/mL TNFα at indicated time and determined the protein levels of RhoB and TNFAIP1 by western blot analysis with β-actin as a loading control. **(B)** Time course of induction of TNFAIP1 and RhoB transcripts upon TNFα treatment in Huh7 cells. Huh7 cells were treated with 20 ng/mL TNFα at indicated time and determined the transcriptional levels of RhoB and TNFAIP1 by qPCR analysis. **(C)** Silencing TNFAIP1 extended the half-life of RhoB in Huh7 cells upon TNFα treatment. Huh7 cells were treated with 20 ng/mL TNFα at indicated time and western blot was used to analyze the RhoB protein levels upon TNFAIP1 knockdown via siRNA silencing with β-actin as a loading control. **(D)** The protein levels were quantified by densitometric analysis and statistical analysis was performed. *P < 0.05, **P < 0.01, and ***P < 0.001 vs. control group; n.s. = not significant.

Given that RhoB participated in the inflammatory response upon TNFα stimulation (Kroon et al., 2013; Marcosramiro et al., 2016), we further determined the role of TNFAIP1-mediated RhoB degradation in TNFα-induced inflammatory response. First, we examined the expression of TNFAIP1 and RhoB in TNFα-stimulated huh7 cells. As shown in Figure 5A, the protein levels of RhoB reached highest at 3 h after TNFα stimulation, and then gradually decreased with the increase of TNFAIP1 expression. Consistently, the mRNA levels of RhoB reached its peak at 1.5 h, accompanied by the transcriptional activation of TNFAIP1 upon TNFα stimulation (Figure 5B). To determine whether the degradation of RhoB after 3 h with TNFα treatment was elicited by TNFAIP1, we examined the turnover rate of RhoB when silencing TNFAIP1. As shown, knockdown of TNFAIP1 significantly blocked the degradation of RhoB after TNFα treatment for 3 h (Figures 5C,D). These results indicated that the turnover of RhoB after TNFα stimulation is regulated by TNFAIP1 in HCC cells.

**FIGURE 6:**
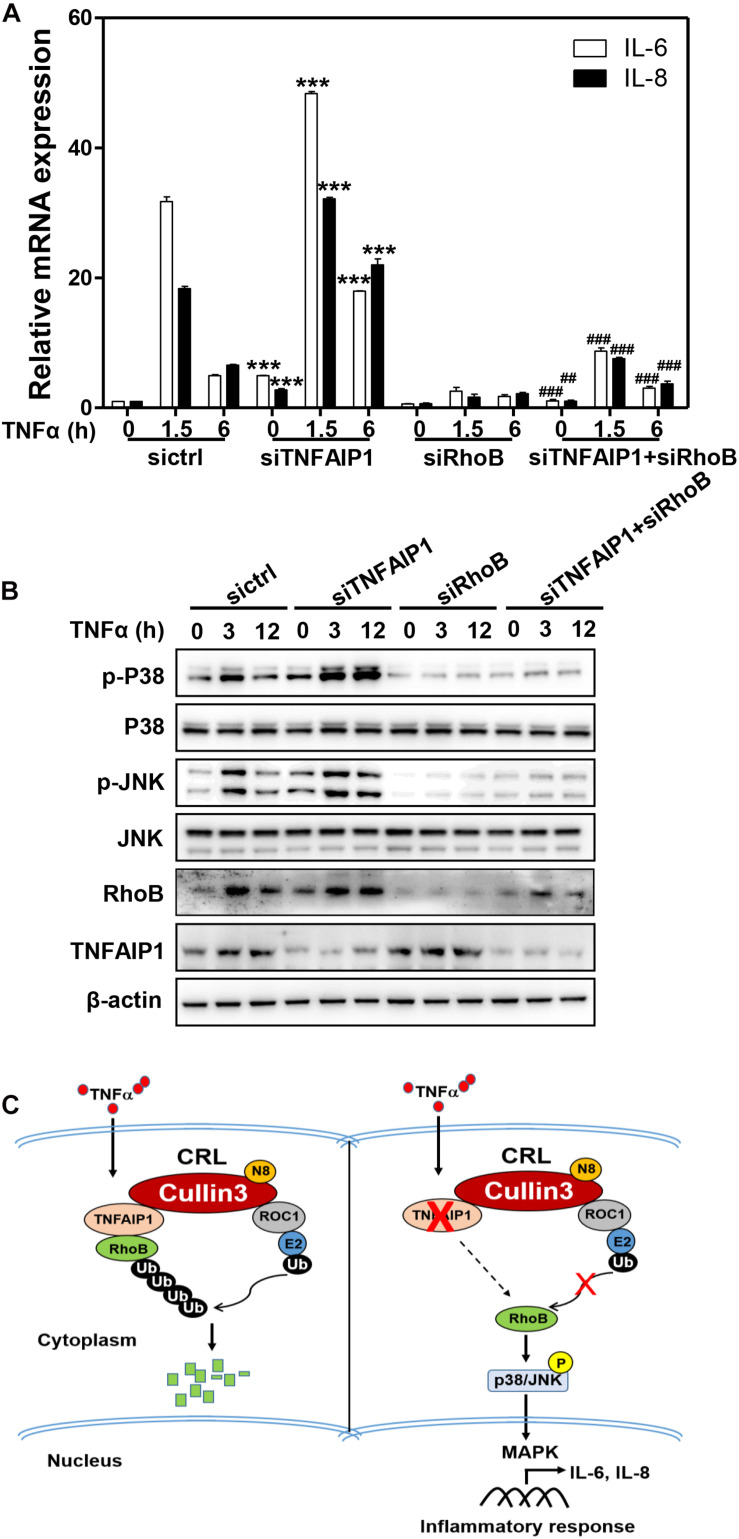
Downregulation of TNFAIP1 triggered inflammatory response in HCC cells via blocking the degradation of RhoB upon TNFα stimulation. **(A)** Silencing TNFAIP1 prolonged the IL-6 and IL-8 transcripts in Huh7 cells upon TNFα treatment, and RhoB knockdown rescued the siTNFAIP1-induced upregulation of IL-6 and IL-8. Huh7 cells were transfected with indicated combination of ctrl (control), TNFAIP1 and RhoB siRNA for 96 h and treated with 20 ng/mL TNFα at indicated time, and the transcriptional levels of IL-6 and IL-8 were determined by qPCR analysis. **(B)** Silencing TNFAIP1 induced the activation of p38/JNK MAPK signaling in Huh7 cells upon TNFα treatment, and knockdown of RhoB reversed the upregulation of phospho-JNK and phospho-p38. Huh7 cells were transfected with indicated combination of ctrl (control), TNFAIP1 and RhoB siRNA for 96 h and treated with 20 ng/mL TNFα at indicated time, and cell lysates were harvested for western blot analysis. **(C)** Working model. Downregulation of TNFAIP1 induced the activation of p38/JNK MAPK signaling pathway and promoted the transcription of IL-6 and IL-8 via blocking RhoB degradation upon TNFα stimulation. ***P < 0.001 vs. control group; ^##^*P* < 0.01 and ^###^*P* < 0.001 vs. siTNFAIP1 group.

Previous studies demonstrated that RhoB plays a pivotal role in the TNFα-induced inflammatory response of endothelial cells and macrophages by activating MAP kinase pathway (Nwariaku et al., 2003; Kroon et al., 2013; Liu et al., 2017). Furthermore, endothelial cells respond to TNFα stimulation by upregulating the expression of pro-inflammatory cytokines, such as IL-6 and IL-8 (Bradley, 2008; Kalliolias and Ivashkiv, 2016). Together with our aforementioned results, we hypothesized that TNFα regulated pro-inflammatory cytokines expression in HCC cells through TNFAIP1-mediated RhoB degradation. As expect, ablation of TNFAIP1 via siRNA silencing markedly upregulated the expression of pro-inflammatory molecules IL-6 and IL-8 in TNFα-stimulated Huh7 cells. Furthermore, the upregulation of IL-6 and IL-8 induced by TNFAIP1 depletion were rescued by simultaneous RhoB knocking down (Figure 6A). We further showed that TNFAIP1 knockdown resulted in the increased levels of phospho-JNK and phospho-p38 upon TNFα stimulation. Rescue experiment results demonstrated that additional RhoB depletion reversed the TNFAIP1 knockdown-induced the accumulations of the phospho-JNK and phospho-p38 (Figure 6B). Taken together, our finding demonstrated that inactivation of TNFAIP1 blocked RhoB degradation, thereby enhancing the inflammatory response of HCC cells induced by activation of MAPK signaling pathway (Figure 6C).

## Discussion

The small GTPase RhoB is critically required for the inflammatory response elicited by pro-inflammatory cytokines (Biro et al., 2014; Zhang et al., 2017). However, the underlying mechanisms of RhoB degradation in inflammatory response remain exclusive. In this study, for the first time, we demonstrated that TNFAIP1 functioned as the novel adaptor connecting RhoB to Cullin3 to target RhoB for ubiquitination and degradation, and TNFα-induced inflammatory response is regulated by TNFAIP1-mediated RhoB expression in HCC cells. Our findings highlight a crucial role of TNFAIP1-induced RhoB degradation in regulating tumor inflammatory response. It is worth noting that Cullin3-KCTD10 complex has also been reported to ubiquitinate RhoB (Kovacevic et al., 2018; Murakami et al., 2019). Therefore, we speculated that KCTD10 and TNFAIP1 might have redundancy or compensation mechanism for RhoB expression. As shown, we found that downregulation of TNFAIP1 or KCTD10 alone induced RhoB upregulation in both Huh7 and HepG2 cells. In addition, knocking down TNFAIP1 and KCTD10 simultaneously resulted in the more accumulation of RhoB compared with TNFAIP1 and KCTD10 knockdown alone (Supplementary Figure 2). Our results suggested that TNFAIP1 and KCTD10 collectively mediated the RhoB expression in HCC cells.

Previous studies found that the pro-inflammatory mediators such as TNFα potently stimulated RhoB expression (Nübel et al., 2004; Rodriguez et al., 2007; Vega and Ridley, 2018). However, it is not clear how RhoB degradation after TNFα stimulation is regulated. In our study, we demonstrated that TNFα induced the transcriptional activation of TNFAIP1 and RhoB at 1.5 h upon TNFα stimulation in HCC cells. Subsequently, RhoB protein levels reached a peak at 3 h, and then gradually decreased to the baseline level with the increase of TNFAIP1 protein expression. While TNFAIP1 was silenced, the half-life of RhoB was significantly delayed after TNFα stimulation for 3 h. Therefore, our findings revealed a previous unknown mechanism by which TNFAIP1 regulated RhoB stability upon TNFα stimulation.

TNFα triggered pro-inflammatory gene expression through the activation of both NF-κB and MAP kinase pathways (Kyriakis and Avruch, 2001; Zelová and Hošek, 2013; Webster and Vucic, 2020). Therefore, we determined the two main signalling cascades activated by TNFα after TNFAIP1 silencing. Consistent with the previous reports that RhoB regulated TNFα-dependent stress-activated MAPKs in endothelial cells (Nwariaku et al., 2003; Kroon et al., 2013). Our data suggested that activation of p38 MAP kinase and JNK by TNFα was critically dependent on TNFAIP1-induced RhoB degradation in HCC cells. However, downregulation of TNFAIP1 had no effect on NF-κB activation upon TNFα stimulation (data not shown). Thus, TNFAIP1-mediated RhoB degradation regulated stress-activated MAPKs in HCC cells. In view of TNFAIP1 was highly expressed in many human cancer cells including lung cancer cells and osteosarcoma cells, and modulated tumorigenesis and cancer cell migration (Zhang et al., 2014; Li et al., 2020). Future study will be performed to elucidate the effect of TNFAIP1-mediated RhoB degradation on tumor inflammatory microenvironment and tumorigenesis.

In summary, our study highlighted a pivotal role of Cullin3-TNFAIP1 complex in mediating RhoB ubiquitination and degradation, and uncovered new mechanisms of CRL3 E3 ligase regulating inflammatory response through TNFAIP1-mediated RhoB degradation. TNFAIP1-RhoB axis played a key role in the regulation of tumor inflammatory microenvironment and could be considered as an attractive target for intervention in human cancers.

## Data Availability Statement

The original contributions presented in the study are included in the article/Supplementary Material, further inquiries can be directed to the corresponding author/s.

## Author Contributions

YZ, HZ, LJ, SW, and LL conceived and designed the experiments. YueL, SW, WZ, and LL performed the experiments and wrote the manuscript. LJ, SW, and LL revised and finalized the manuscript. LC, YP, and YupL contributed to critical experiment assistances. JX and YJ performed statistical analysis of the data. All authors read and approved the final manuscript.

## Conflict of Interest

The authors declare that the research was conducted in the absence of any commercial or financial relationships that could be construed as a potential conflict of interest.

## Supplementary Material

Supplementary Figure 1 Downregulation of TNFAIP1 did not affect the protein level of Cullin3 in Huh7 or HepG2 cells. Cells were transfected with ctrl (control), TNFAIP1-1 or TNFAIP1-2 siRNA for 96 h, and cell lysates were harvested for western blot analysis.

Supplementary Figure 2 Knocking down TNFAIP1 and KCTD10 simultaneously resulted in the more accumulation of RhoB compared with TNFAIP1 and KCTD10 knockdown alone. Huh7 and HepG2 cells were transfected with indicated combination of ctrl (control), TNFAIP1 and KCTD10 siRNA for 96 h and cell lysates were harvested for western blot analysis.
