# Supplementary figures and images for: Cullin3-TNFAIP1 E3 Ligase Controls Inflammatory Response in Hepatocellular Carcinoma Cells via Ubiquitination of RhoB
Source: Front Cell Dev Biol. 2021 Jan 21;9:617134. doi: 10.3389/fcell.2021.617134 (PMC7859282; doi:10.3389/fcell.2021.617134)

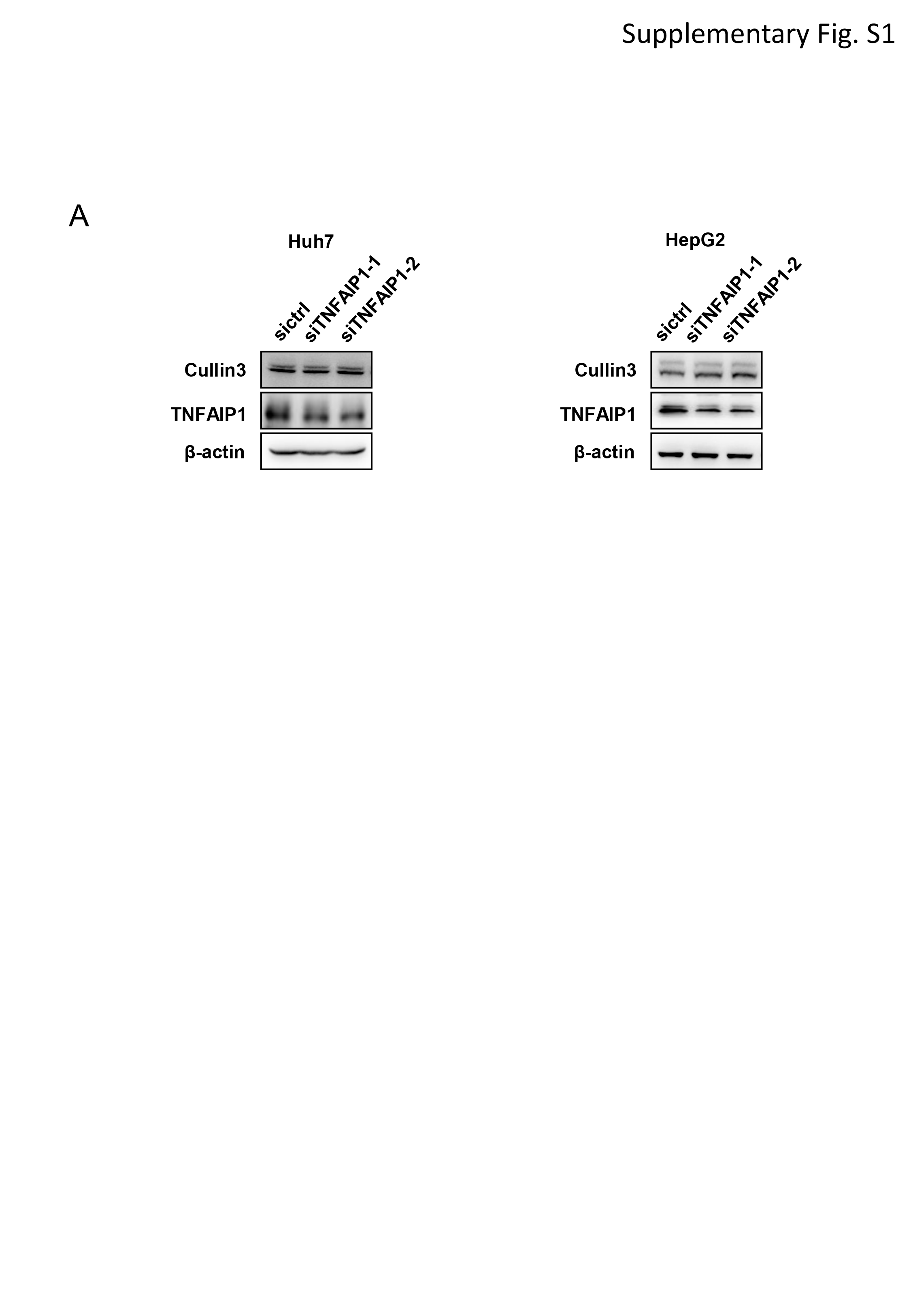

Supplement: Supplementary Figure 1 — Downregulation of TNFAIP1 did not affect the protein level of Cullin3 in Huh7 or HepG2 cells. Cells were transfected with ctrl (control), TNFAIP1-1 or TNFAIP1-2 siRNA for 96 h, and cell lysates were harvested for western blot analysis. [file Image_1.TIF]

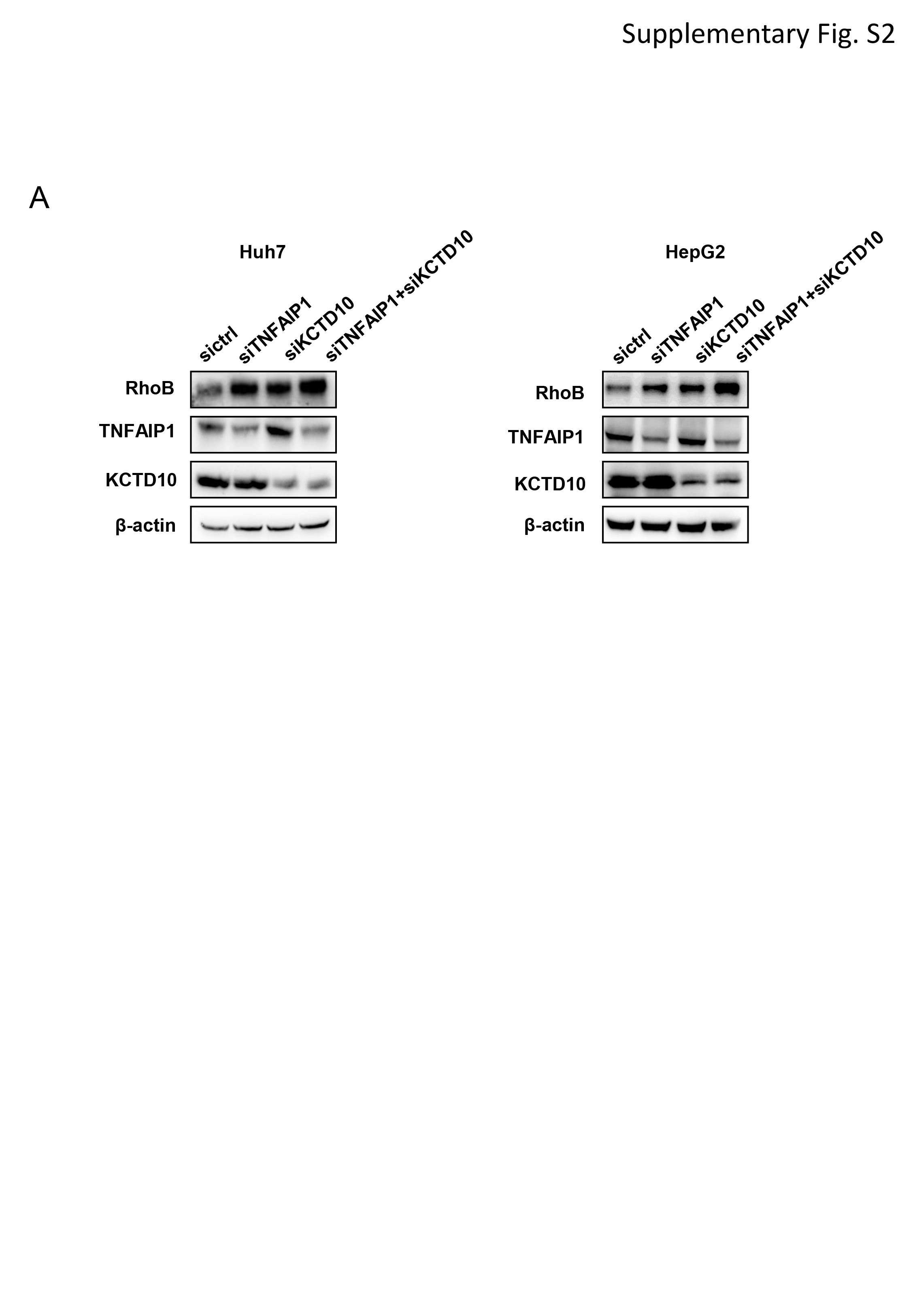

Supplement: Supplementary Figure 2 — Knocking down TNFAIP1 and KCTD10 simultaneously resulted in the more accumulation of RhoB compared with TNFAIP1 and KCTD10 knockdown alone. Huh7 and HepG2 cells were transfected with indicated combination of ctrl (control), TNFAIP1 and KCTD10 siRNA for 96 h and cell lysates were harvested for western blot analysis. [file Image_2.TIF]
